# Stimulant-Related Emergencies in Older Adults

**DOI:** 10.1001/jamanetworkopen.2025.12860

**Published:** 2025-05-29

**Authors:** Jonathan S. Zipursky, Rachela Smith, Ania Sarnocinska, Jagadish Rangrej, Inas Riyaz Mohammed, Saad Rais, Kamil Malikov, Michael P. Hillmer, Nathan M. Stall

**Affiliations:** 1 Department of Medicine, University of Toronto, Toronto, Ontario, Canada; 2 Division of General Internal Medicine, Sunnybrook Health Sciences Centre, Toronto, Ontario, Canada; 3 Institute of Health Policy, Management, and Evaluation, University of Toronto, Toronto, Ontario, Canada; 4 Michael DeGroote School of Medicine, Hamilton, Ontario, Canada; 5 Digital Analytics Strategy Division, Ontario Ministry of Health, Toronto, Ontario, Canada; 6 Division of General Internal Medicine and Geriatrics, Sinai Health and University Health Network, Toronto, Ontario, Canada; 7 Women’s Age Lab, Women’s College Hospital, Toronto, Ontario, Canada

## Abstract

This cohort study examines annual rates of stimulant-related emergency department visits among older adults.

## Introduction

Most data on substance use in older adults have focused on alcohol, opioids, and cannabis, yet there is a growing concern about stimulant use.^1^ The majority of people born between 1946 and 1964 (commonly referred to as “Baby Boomers”) are now over the age of 65 years, and this generation has historically used substances at higher rates than previous generations. While stimulant-related health care visits have increased among all adults over the last 2 decades,^2,3^ there are limited population-based data on stimulant-related emergency department visits specific to older adults.

## Methods

We conducted a population-based retrospective cohort study examining annual stimulant-related emergency department (ED) visits in older adults (aged 66 years or more) between January 2010 and December 2023 in Canada’s most populous province, Ontario (16 million residents). We used linked administrative data from the Ontario Ministry of Health; the primary outcome was the annual rate (per 100 000 population) of ED visits in older adults for stimulant related emergencies. We identified ED visits where *International Statistical Classification of Diseases and Related Health Problems, Tenth Revision (ICD-10)* codes corresponding to mental and behavioral disorders or poisoning from cocaine and/or stimulants were listed as the main or contributing reason for the ED visit (eAppendix in Supplement 1). We allowed participants to contribute multiple ED visits to the analysis. We also examined sex, age, medical comorbidities commonly associated with prescription stimulant use, and dispensations for stimulant prescriptions. Data on race and ethnicity were unavailable. All statistical analyses were conducted using SAS version 9.4 (SAS Institute). The study was approved by the Sunnybrook Health Sciences Research Ethics Board and was exempt from informed consent requirements because data were deidentified. We followed the Strengthening the Reporting of Observational Studies in Epidemiology (STROBE) reporting guideline for cross-sectional studies (eAppendix in Supplement 1).

## Results

Between 2010 and 2023 there were 1247 stimulant-related ED visits in older adults; median (IQR) age was 69 years (67-73 years), 934 (74.9%) were male, and 579 (46.4%) were in the lowest income quintile (Table). Of the stimulant-related visits, 806 (64.6%) were related to cocaine, 412 (33.0%) to other stimulants, and 29 (2.3%) to both. Rates of stimulant-related ED visits in older individuals increased from 1.4 to 12.6 per 100 000 from 2010 to 2023 (Figure). Among stimulant-related ED visits, 20 (1.6%) were preceded by a prescription fill for a psychostimulant drug within 90 days. In addition, 23 visits (1.8%) occurred in patients with Parkinson disease, 8 (0.6%) with attention deficit hyperactivity disorder, and 34 (2.7%) in people living with dementia. Moreover, 216 visits (17.3%) occurred in patients with a history of psychosis, 687 (55.1%) with substance use disorder, 415 (33.3%) with mood disorders, and 662 (53.1%) with anxiety disorders.

**Table.** Characteristics of Older Adults With Stimulant-Related Emergency Department Visits

Characteristics	Visits, No. (%) (N = 1247)^a^
Age, median (IQR), y	69 (67-73)
Age group, y	
66-74	1031 (82.7)
75-84	166 (13.3)
≥85	50 (4.0)
Sex	
Female	313 (25.1)
Male	934 (74.9)
Location of residence	
Rural	102 (8.2)
Urban	1093 (87.7)
Neighborhood income, quintile	
1st (lowest)	579 (46.4)
2nd	247 (19.8)
3rd	196 (15.7)
4th	87 (7.0)
5th (highest)	86 (6.9)
Prescription for psychostimulant^b^	20 (1.6)
Narcolepsy	0
Parkinson disease	23 (1.8)
ADHD	8 (0.6)
Dementia	34 (2.7)
Mental health	
Psychotic disorder	216 (17.3)
Substance use disorder	687 (55.1)
Mood disorder	415 (33.3)
Anxiety disorder	662 (53.1)

Abbreviation: ADHD, attention deficit hyperactivity disorder.

^a^ Missing data included 52 visits missing for location of residence and neighborhood income.

^b^ Prescription fill for methylphenidate, lisdexamfetamine, dextroamphetamine, or mixed amphetamine salts in the 90 days prior to index date.

**Figure.**
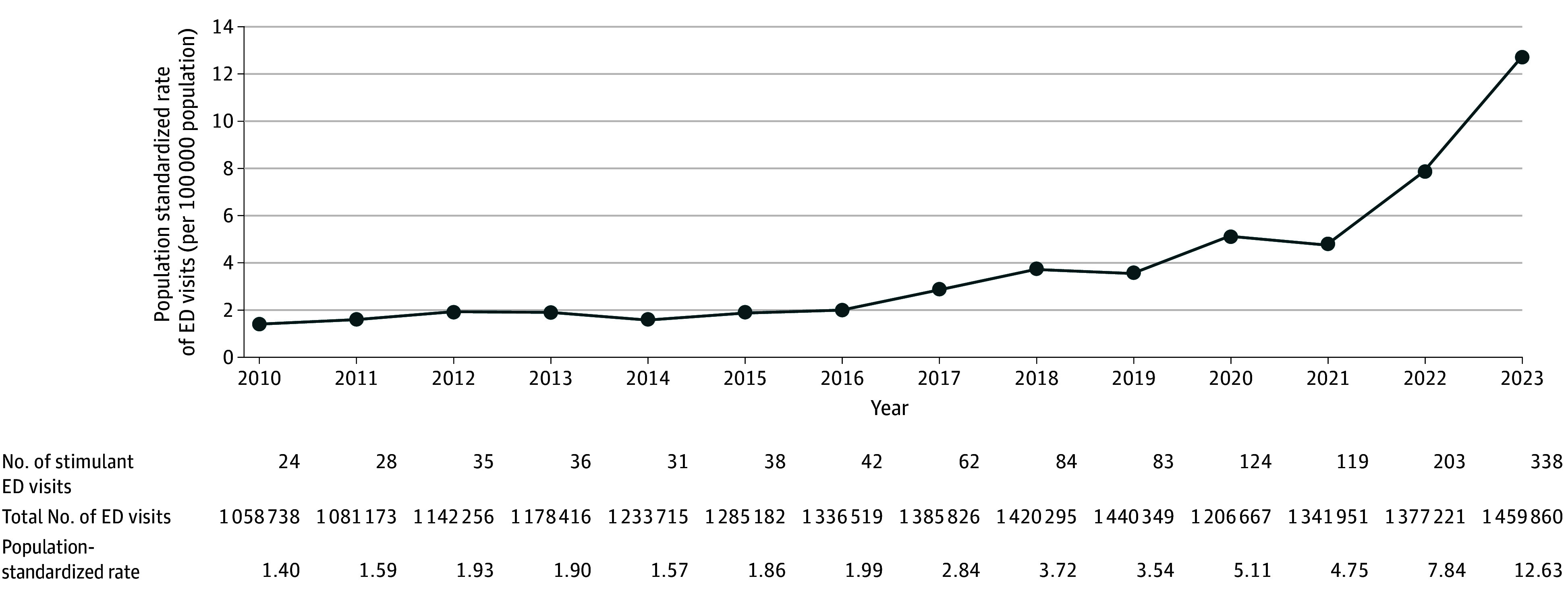
Stimulant-Related Emergency Department (ED) Visits in Older Adults in Ontario, Canada From 2010 to 2023

## Discussion

We observed a 9-fold increase in annual stimulant-related ED visits among Ontario older adults between 2010 and 2023. Few patients experiencing stimulant-related ED visits filled prescriptions for psychostimulants or had conditions that are commonly treated with stimulants, and more than two-thirds of ED visits were related to cocaine use. The observed trends may be attributable to increases in recreational stimulant use among older adults (eg, cocaine, amphetamines, ecstasy), the rising prevalence of high potency stimulants in the unregulated drug supply, misappropriation of prescribed stimulants, and growing social acceptance of psychoactive substances. Older adults may be at high risk of adverse drug events from stimulants due to age-related physiological changes, polypharmacy, and multimorbidity.^4^

Study limitations include restricting data to ED visits, which may underestimate the number of stimulant-related poisonings and mental and behavioral disorders.^5^ Some older adults using stimulants may have avoided hospital care or sought care in other settings. Finally, reasons for stimulant use were unavailable.

While the absolute number of ED visits attributable to stimulant toxicity remained low, our study highlights an increasing trend of stimulant-related harms among older adults, especially in lower income men and those with mental health and addictions disorders. Prescription and nonprescription stimulant use have been linked to an increased risk of adverse health outcomes in older adults including hypertension, stroke, myocardial infarction, and arrhythmia.^6^ Overall there is a need for increased stimulant use screening among older adults and enhanced clinical engagement to minimize stimulant-related harms.^4^

## Notes

Supplement 1.
eMethods.

eReferences

Supplement 2.
Data Sharing Statement
