# Supplementary material for: Stimulant-Related Emergencies in Older Adults
Source: JAMA Netw Open. 2025 May 29;8(5):e2512860. doi: 10.1001/jamanetworkopen.2025.12860 (PMC12123467; doi:10.1001/jamanetworkopen.2025.12860)
Supplement: Supplement 1. — eMethods. eReferences [file jamanetwopen-e2512860-s001.pdf]

Supplemental Online Content

Zipursky JS, Smith R, Sarnocinska A, et al. Stimulant-related emergencies in older adults. *JAMA Netw Open.* 2025;8(5):e2512860.
doi:10.1001/jamanetworkopen.2025.12860

eMethods

eReferences

This supplemental material has been provided by the authors to give readers additional information about their work.

© 2025 Zipursky JS et al. *JAMA Network Open*.

eMethods

1.1 Codes for Stimulant-Related Emergency Department Visits

ICD10 Code [1–4]	Description
F14	Mental and behavioural disorders due to use of cocaine
F15	Mental and behavioural disorders due to use of other stimulants, including caffeine
R78.2	Finding of cocaine in the blood
T40.5	Cocaine poisoning
T43.60	Poisoning by unspecific psychostimulants
T43.62	Poisoning by amphetamines
T43.63	Poisoning by methylphenidate
T43.64	Poisoning by ecstasy
T43.65	Poisoning by methamphetamines
T43.68	Poisoning by other specified psychostimulants with abuse potential
T43.69	Poisoning by other psychostimulants
Y49.7	Psychostimulants with abuse potential

1.2 Codes for Narcolepsy

Algorithm[5–9]: ≥1 claim including a diagnosis code of narcolepsy in the 2 years prior to index date

	Code	Description
ICD9/OHIP	347	Cataplexy and narcolepsy
ICD10	G47.4	Narcolepsy and cataplexy

1.3 Codes for Parkinson's Disease (PD)

Algorithm[10]: PD defined as three physician billing codes for PD (diagnosis code: 332) in 2 years each separated by at least 30 days using physician billing data

	Code	Description
ICD9/OHIP	332	Central Nervous System – Parkinson's Disease

1.4 Codes for Attention Deficit Hyperactivity Disorder (ADHD)

Algorithm[11]: ADHD defined as any claim including a diagnosis code (outpatient, inpatient) in the 2 years prior to index date

	Code	Description
ICD9/OHIP	314	Hyperkinetic syndrome of childhood

© 2025 Zipursky JS et al. *JAMA Network Open*.

ICD10	F90	Attention-deficit hyperactivity disorders

1.5 Codes for Dementia

Patients living with dementia were identified by using a validated administrative algorithm (1 hospitalization, 3 physician claims, or 1 drug, which was earliest)[12,13]:

- One hospitalization billing including any diagnosis of dementia; or
- Three physician claims in two years separated by at least 30 days including a diagnosis of dementia; or
- One prescription filled for an Alzheimer's Disease Related Dementias medication (cholinesterase inhibitor: donepezil, galantamine, or rivastigmine)

1.6 Codes for Mental Health Disorders

Algorithm [14]: having ≥1 service utilization visits including any of the following diagnostic codes in the 2 years before index.

Diagnostic Category	Outpatient OHIP Codes	ED Visits NACRS Codes	Hospitalization DAD Codes	Hospitalization OHMRS Codes
Psychotic Disorders	295, 297, 298	DX10CODE 1: F06.0-2, F20, F22-F29, F53.1<br><br>***Prior to 2016/2017: F20 (excluding F20.4), F22, F23, F24, F25, F28, F29, F53.1	DX10CODE 1: F06.0-2, F20, F22-F29, F53.1<br><br>***Prior to 2016/2017: DX10CODE 1: F20 (excluding F20.4), F22, F23, F24, F25, F28, F29, F53.1	Pre-2016 Codes: AXIS1_DSM4CODE_DISCH1 = 295.x (all 295 codes), 297.x (all 297 codes), 298.x (all 298 codes).<br><br>2016/17 – 2018/19 Codes: DSM5CODE_DISCH1 = 293.81, 293.82, 295.x (all 295 codes), 297.x (all 297 codes), 298.x (all 298 codes).<br><br>Post-2019 Codes: ICD10CMCODE_DISCH1 = F20.81, F20.9, F22, F23, F25, F06.0/1/2, F28, F29.
Overall Mood Disorders	296, 311	DX10CODE 1: F06.3, F30.x-F34.x, F38.x, F39.x, F53.0<br><br>***Prior to 2016/2017: DX10CODE1 = F30, F31, F32, F33, F34, F38, F39, F53.0	DX10CODE 1: F06.3, F30.x-F34.x, F38.x, F39.x, F53.0<br><br>***Prior to 2016/2017: DX10CODE1 = F30, F31, F32, F33, F34, F38, F39, F53.0	Pre-2016 Codes: AXIS1_DSM4CODE_DISCH1 = 296.x (all 296 codes), 300.4x, 301.13, 311.x<br><br>2016/17 – 2018/19 Codes: DSM5CODE_DISCH1 = 293.83, 296.x (all 296 codes), 300.4x, 301.13, 311.x, 625.4.<br><br>Post-2019 Codes: ICD10CMCODE_DISCH1 = F06.3, F31, F32, F33, F34,
Anxiety and Related Disorders	300	DX10CODE 1: F06.4, F40, F41, F42.x, F43.x, F45.2, F63.3, F93.0-2, F94.0-2<br><br>***Prior to 2016/2017: DX10CODE1 = F40, F41, F42, F43, F48.8, F48.9; F93.1, F93.2	DX10CODE 1: F06.4, F40, F41, F42.x, F43.x, F45.2, F63.3, F93.0-2, F94.0-2<br><br>***Prior to 2016/2017: DX10CODE1 = F40, F41, F42, F43, F48.8, F48.9; F93.1, F93.2	Pre-2016 Codes: AXIS1_DSM4CODE_DISCH1 = 300, 300.0x, 300.2x, 300.3x, 308.3x, 309.0x, 309.24, 309.28, 309.3x, 309.4x, 309.8x, 309.9x.<br><br>2016/17 – 2018/19 Codes: DSM5CODE_DISCH1 = 293.84, 300, 300.0x, 300.2x, 300.3x, 300.7x, 308.3x, 309, 309.0x, 309.21, 309.24, 309.28, 309.3, 309.4x, 309.81,

© 2025 Zipursky JS et al. *JAMA Network Open*.

				309.89, 309.9x, 312.39, 313.23, 313.89, 698.4x.<br><br>Post-2019 Codes: ICD10CMCODE_DISCH1 = F06.4, F06.8, F40.0x, F40.1x. F40.2x, F41.0x/1x, F41.8x/9x, F42.2x, F42.3, F42.4, F42.8, F42.9, F43.0, F43.1. F43.2, F43.8/9, F45.2, F63.3, F93.0, F94.0, F94.1/2.
Substance and Alcohol Use Disorders	291, 292, 303, 304	DX10CODE 1: F10-19, F55,<br><br>***Prior to 2016/2017: DX10CODE1 = F55, F10 to F19	DX10CODE 1: F10-19, F55,<br><br>***Prior to 2016/2017: DX10CODE1 = F55, F10 to F19	Pre-2016 Codes: AXIS1_DSM4CODE_DISCH1 = 291.x (all 291 codes, excluding 291.82), 292.x (all 292 codes, excluding 292.85), 303.x (all 303 codes), 304.x (all 304 codes), 305.x (all 305 codes).<br><br>2016/17 – 2018/19 Codes: DSM5CODE_DISCH1 = 291.x (all 291 codes), 292.x (all 292 codes), 303.x (all 303 codes), 304.x (all 304 codes), 305.x.<br><br>Post-2019 Codes: ICD10CMCODE_DISCH1 = F10.x-F19.x, Z72.0.

1.7 Identification of psychostimulant prescriptions

Patients who filled a psychostimulant prescription were identified with a 90-day look back from the index date (not including index date) for filled prescriptions of methylphenidate, mixed amphetamine salts, dextroamphetamine, or lisdexamfetamine using drug identification numbers (DINs).

© 2025 Zipursky JS et al. *JAMA Network Open*.

eReferences

1. Rowe CL, Santos GM, Kornbluh W, Bhardwaj S, Faul M, Coffin PO. Using ICD-10-CM codes to detect illicit substance use: A comparison with retrospective self-report. *Drug Alcohol Depend*. 2021;221:108537. doi:10.1016/j.drugalcdep.2021.108537
2. Shearer RD, Shippee ND, Winkelman TNA. Characterizing trends in methamphetamine-related health care use when there is no ICD code for "methamphetamine use disorder." *J Subst Abuse Treat*. 2021;127:108369. doi:10.1016/j.jsat.2021.108369
3. Centers for Disease Control and Prevention. Drug Use-Associated Hospital Encounters Involving COVID-19 by Month From Selected Hospitals. September 3, 2024. Accessed October 17, 2024. https://www.cdc.gov/nchs/dhcs/drug-use/covid19.htm
4. Federal, provincial, and territorial Special Advisory Committee on the Epidemic of Opioid Overdoses. Opioid- and Stimulant-related Harms in Canada. September 2023. Accessed October 19, 2024. https://health-infobase.canada.ca/substance-related-harms/opioids-stimulants/
5. Carls G, Reddy SR, Broder MS, et al. Burden of disease in pediatric narcolepsy: a claims-based analysis of health care utilization, costs, and comorbidities. *Sleep Med*. 2020;66:110-118. doi:10.1016/j.sleep.2019.08.008
6. Kallweit U, Nilius G, Trümper D, Vogelmann T, Schubert T. Prevalence, incidence, and health care utilization of patients with narcolepsy: a population-representative study. *Journal of Clinical Sleep Medicine*. 2022;18(6):1531-1537. doi:10.5664/jcsm.9910
7. Crispo JAG, Liu L, Bach P, et al. Amphetamine-Related Emergency Department Visits in Ontario, Canada, 2003-2020. *The Canadian Journal of Psychiatry*. 2023;68(11):838-849. doi:10.1177/07067437231158933
8. Jolley RJ, Liang Z, Peng M, et al. Identifying Cases of Sleep Disorders through International Classification of Diseases (ICD) Codes in Administrative Data. *Int J Popul Data Sci*. 2018;3(1). doi:10.23889/ijpds.v3i1.448
9. Weibel D, Sturkenboom M, Black S, et al. Narcolepsy and adjuvanted pandemic influenza A (H1N1) 2009 vaccines - Multi-country assessment. *Vaccine*. 2018;36(41):6202-6211. doi:10.1016/j.vaccine.2018.08.008
10. Maclagan LC, Marras C, Sewell IJ, et al. Trends in health service use among persons with Parkinson's disease by rurality: A population-based repeated cross-sectional study. *PLoS One*. 2023;18(5):e0285585. doi:10.1371/journal.pone.0285585
11. Butt DA, Jaakkimainen L, Tu K. Prevalence and Incidence Trends of Attention Deficit/Hyperactivity Disorder in Children and Youth Aged 1–24 Years in Ontario, Canada: A Validation Study of Health Administrative Data Algorithms: Tendances de la prévalence et de l'incidence du trouble de déficit de l'attention/hyperactivité chez les enfants et les jeunes âgés de 1 à 24 ans, en Ontario, Canada: une étude de validation des algorithmes de données administratives de santé. *The Canadian Journal of Psychiatry*. 2024;69(5):326-336. doi:10.1177/07067437231213553
12. Stall NM, Shi S, Malikov K, et al. Edible Cannabis Legalization and Cannabis Poisonings in Older Adults. *JAMA Intern Med*. Published online May 20, 2024. doi:10.1001/jamainternmed.2024.1331
13. Jaakkimainen RL, Bronskill SE, Tierney MC, et al. Identification of Physician-Diagnosed Alzheimer's Disease and Related Dementias in Population-Based Administrative Data: A

© 2025 Zipursky JS et al. *JAMA Network Open*.

Validation Study Using Family Physicians' Electronic Medical Records. *Journal of Alzheimer's Disease*. 2016;54(1):337-349. doi:10.3233/JAD-160105
14. Kurdyak P, Lebenbaum M, Patrikar A, et al. SARS-CoV-2 vaccination prevalence by mental health diagnosis: a population-based cross-sectional study in Ontario, Canada. *CMAJ Open*. 2023;11(6):E1066-E1074. doi:10.9778/cmajo.20220210

© 2025 Zipursky JS et al. *JAMA Network Open*.
